# Supplementary material for: Predicting referral need for febrile children in low-resource community settings in South and Southeast Asia
Source: Nat Med. 2026 Apr 29;32(5):1907–16. doi: 10.1038/s41591-026-04338-1 (PMC13190324; doi:10.1038/s41591-026-04338-1)
Supplement: Supplementary file 1 — Supplementary Tables 1−15. [file 41591_2026_4338_MOESM1_ESM.pdf]

nature medicine

Supplementary information

https://doi.org/10.1038/s41591-026-04338-1

Predicting referral need for febrile children in low-resource community settings in South and Southeast Asia

In the format provided by the authors and unedited

Predicting which febrile children need referral in low-resource community settings in South and Southeast Asia

SUPPLEMENTARY APPENDIX

Table of Contents

1. *Table S1: Microbiological causes of infection in the derivation and validation cohorts.* *2*
2. *Table S2: Candidate predictors entered into the prediction models.* *3*
3. *Table S3: Variable selection and performance of the clinical-biomarker models when each biomarker was included in turn alongside the candidate clinical predictors.* *4*
4. *Table S4: Variable selection and performance of the clinical-biomarker models when each biomarker was included in turn alongside the candidate clinical predictors and* SpO_2. .... *5*
5. *Table S5: Variable selection and performance of the clinical-biomarker models when each biomarker was included in turn alongside the candidate clinical predictors, excluding the northern Viet Nam site.* *6*
6. *Tables S6: Logistic regression equations for the four clinical prediction models.* *7*
7. *Table S7: Categorical outcome scale.* *9*
8. *Table S8: Characteristics of participants who developed severe disease, stratified by whether they were identified by each prediction model (derivation and validation cohorts pooled)* *10*
9. *Table S9: Variation in predicted cost-effectiveness with increasing referral costs.* *12*
10. *Table S10: Methodology for collection of clinical parameters.* *13*
11. *Table S11: Existing evidence supporting selection of endothelial and immune activation markers.* *14*
12. *Table S12: Laboratory procedures for biomarker quantification.* *15*
13. *Table S13: Derivation of site-specific outpatient weights.* *16*
14. *Table S14: Parameters for cost-effectiveness analyses.* *18*
15. *Table S15: TRIPOD checklist.* *19*

1. **Table S1: Microbiological causes of infection in the derivation and validation cohorts.**

**Characteristic**	**Overall** N = 3,405[1]	**Derivation cohort** N = 2,581[1]	**Validation cohort** N = 824[1]
Respiratory syncytial virus	429 / 2,861 (15%)	349 / 2,209 (16%)	80 / 652 (12%)
Dengue	109 / 3,289 (3.3%)	107 / 2,474 (4.3%)	2 / 815 (0.2%)
Influenza A	87 / 2,861 (3.0%)	69 / 2,209 (3.1%)	18 / 652 (2.8%)
Influenza B	59 / 2,861 (2.1%)	59 / 2,209 (2.7%)	0 / 652 (0%)
Human metapneumovirus	65 / 2,209 (2.9%)	65 / 2,209 (2.9%)	0 / 0
SARS-CoV-2	81 / 2,861 (2.8%)	74 / 2,209 (3.3%)	7 / 652 (1.1%)
Bacteraemia*	19 / 1,192 (1.6%)	12 / 781 (1.5%)	7 / 411 (1.7%)
Chikungunya	47 / 3,289 (1.4%)	4 / 2,474 (0.2%)	43 / 815 (5.3%)
Bordetella parapertussis	8 / 2,209 (0.4%)	8 / 2,209 (0.4%)	0 / 0
Zika	8 / 3,289 (0.2%)	1 / 2,474 (<0.1%)	7 / 815 (0.9%)
Rickettsia spp.	6 / 3,128 (0.2%)	5 / 2,313 (0.2%)	1 / 815 (0.1%)
Leptospirosis spp.	4 / 3,128 (0.1%)	4 / 2,313 (0.2%)	0 / 815 (0%)
Mycoplasma pneumoniae	3 / 2,209 (0.1%)	3 / 2,209 (0.1%)	0 / 0
Chlamydia pneumoniae	3 / 2,209 (0.1%)	3 / 2,209 (0.1%)	0 / 0
Orientia tsutsugamushi	3 / 3,128 (<0.1%)	1 / 2,313 (<0.1%)	2 / 815 (0.2%)
Bordetella pertussis	1 / 2,209 (<0.1%)	1 / 2,209 (<0.1%)	0 / 0

[1]n / N (%)

*Bacteraemia diagnosed by blood culture: *Staphylococcus aureus*, n = 5; *Escherichia coli*, n = 3; *Acinetobacter* spp., n = 2; *Salmonella* spp., n = 2; Other, n = 7 (*Campylobacter jejuni*, n = 1; *Enterobacter* spp., n = 1; *Enterococcus faecalis*, n = 1; *Haemophilus influenzae*, n = 1; *Streptococcus pneumoniae*, n = 1; *Streptococcus pyogenes*, n = 1; *Vibrio* spp., n = 1). In Cambodia (validation cohort), respiratory samples were tested for SARS-CoV-2, influenza A, influenza B, and RSV. In Indonesia (part of the derivation cohort), PCR testing was performed for Zika, dengue, and chikungunya. When feasible, the study supported collection of blood cultures at the discretion of the treating clinical team.

2. Table S2: Candidate predictors entered into the prediction models.

Parameter (units)	Method / Definition	Handling
Age (months)	Interview with caregiver	Continuous
Mid-upper arm circumference (mm)	*Médecins Sans Frontières* traffic light MUAC tape	Continuous
Heart rate (bpm)	Massimo Rad-5v pulse oximeter with paediatric and neonatal probes	Continuous
Respiratory rate (bpm)	Manual count for 60 seconds using clicker counter and timer	Continuous
Axillary temperature (°C)	Digital thermometer: operating range 32.0-42.9°C; accuracy ± 0.1°C	Continuous
Mental state (AVPU scale)	Alert (A) vs. not alert (V or P or U)	Binary
Capillary refill time	Pressure applied to sternum for five seconds; prolonged > 2 seconds	Binary
Recent hospitalisation	Interview with caregiver; overnight admission within last 6 months	Binary
Convulsions	Interview with caregiver; period of unresponsiveness followed by stiffening of the limbs and/or repetitive, rhythmic movements of a part of the body	Binary
Prostration	Inability to feed, sit, stand, or walk when previously able	Binary
Intractable vomiting	Interview with caregiver; vomited after every feed or drink in last 12h	Binary
Oxygen saturation in room air (%)	Massimo Rad-5v pulse oximeter with paediatric and neonatal probes	Continuous
ANG-1 (pg/ml)	Simple Plex Ella microfluidic platform (EDTA-plasma)	Continuous
ANG-2 (pg/ml)	Simple Plex Ella microfluidic platform (EDTA-plasma)	Continuous
sFLT-1 (pg/ml)	Simple Plex Ella microfluidic platform (EDTA-plasma)	Continuous
CHI3L1 (ng/ml)	Simple Plex Ella microfluidic platform (EDTA-plasma)	Continuous
CRP (mg/l)	Simple Plex Ella microfluidic platform (EDTA-plasma)	Continuous
IL-1ra (pg/ml)	Simple Plex Ella microfluidic platform (EDTA-plasma)	Continuous
IL-6 (pg/ml)	Simple Plex Ella microfluidic platform (EDTA-plasma)	Continuous
IL-8 (pg/ml)	Simple Plex Ella microfluidic platform (EDTA-plasma)	Continuous
IL-10 (pg/ml)	Simple Plex Ella microfluidic platform (EDTA-plasma)	Continuous
IP-10 (pg/ml)	Simple Plex Ella microfluidic platform (EDTA-plasma)	Continuous
PCT (ng/ml)	Simple Plex Ella microfluidic platform (EDTA-plasma)	Continuous
sTNF-R1 (pg/ml)	Simple Plex Ella microfluidic platform (EDTA-plasma)	Continuous
sTREM1 (pg/ml)	Simple Plex Ella microfluidic platform (EDTA-plasma)	Continuous
suPAR (ng/ml)	suPARnostic ELISA (EDTA-plasma)	Continuous
Lactate (mmol/l)	LACT2 platform (fluoride-oxalate plasma)	Continuous
Glucose (mmol/l)	GLUC3 platform (fluoride-oxalate plasma)	Continuous
Hb (g/dL)	Local hospital laboratory platform (EDTA whole blood)	Continuous

3. **Table S3: Variable selection and performance of the *clinical-biomarker models* when each biomarker was included in turn alongside the candidate clinical predictors.**

	sTREM1	CRP	PCT	ANG-2	sTNF-R1	sFLT-1	Hb	IL-6	IL-1ra	CHI3L1	suPAR	IL-10	IL-8	IP-10	ANG-1	Glucose	Lactate
**Age (months)**	Red	Red	Red	Red	Red	Red	Red	Red	Red	Red	Red	Red	Red	Red	Red	Red	Red
**MUAC (mm)**	Red	Red	Red	Red	Red	Red	Red	Red	Red	Red	Red	Red	Red	Red	Red	Red	Red
**Heart rate (bpm)**	Green	Green	Green	Green	Green	Green	Green	Green	Green	Green	Green	Green	Green	Green	Green	Green	Green
**Respiratory rate (bpm)**	Green	Green	Green	Green	Green	Green	Green	Green	Green	Green	Green	Green	Green	Green	Green	Green	Green
**Temperature (°C)**	Red	Green	Red	Red	Red	Red	Red	Red	Red	Red	Red	Red	Red	Red	Red	Red	Red
**Altered mental state**	Green	Green	Green	Green	Green	Green	Green	Green	Green	Green	Green	Green	Green	Green	Green	Green	Green
**Prolonged CRT**	Red	Green	Green	Green	Green	Green	Green	Green	Green	Green	Green	Green	Green	Green	Green	Green	Green
**Recent hospitalisation**	Red	Red	Red	Red	Red	Red	Red	Red	Red	Red	Red	Red	Red	Red	Red	Red	Red
**Prostration**	Green	Green	Green	Green	Green	Green	Green	Green	Green	Green	Green	Green	Green	Green	Green	Green	Green
**Intractable vomiting**	Green	Green	Green	Green	Green	Green	Green	Green	Green	Green	Green	Green	Green	Green	Green	Green	Green
**Convulsions**	Red	Red	Red	Red	Red	Red	Red	Red	Red	Red	Red	Red	Red	Red	Red	Red	Red
**sTREM1 (pg/ml)**	Green	Yellow	Yellow	Yellow	Yellow	Yellow	Yellow	Yellow	Yellow	Yellow	Yellow	Yellow	Yellow	Yellow	Yellow	Yellow	Yellow
**CRP (mg/l)**	Yellow	Green	Yellow	Yellow	Yellow	Yellow	Yellow	Yellow	Yellow	Yellow	Yellow	Yellow	Yellow	Yellow	Yellow	Yellow	Yellow
**PCT (ng/ml)**	Yellow	Yellow	Green	Yellow	Yellow	Yellow	Yellow	Yellow	Yellow	Yellow	Yellow	Yellow	Yellow	Yellow	Yellow	Yellow	Yellow
**ANG-2 (pg/ml)**	Yellow	Yellow	Yellow	Green	Yellow	Yellow	Yellow	Yellow	Yellow	Yellow	Yellow	Yellow	Yellow	Yellow	Yellow	Yellow	Yellow
**sTNF-R1 (pg/ml)**	Yellow	Yellow	Yellow	Yellow	Green	Yellow	Yellow	Yellow	Yellow	Yellow	Yellow	Yellow	Yellow	Yellow	Yellow	Yellow	Yellow
**sFLT-1 (pg/ml)**	Yellow	Yellow	Yellow	Yellow	Yellow	Green	Yellow	Yellow	Yellow	Yellow	Yellow	Yellow	Yellow	Yellow	Yellow	Yellow	Yellow
**Hb (g/dl)**	Yellow	Yellow	Yellow	Yellow	Yellow	Yellow	Green	Yellow	Yellow	Yellow	Yellow	Yellow	Yellow	Yellow	Yellow	Yellow	Yellow
**IL-6 (pg/ml)**	Yellow	Yellow	Yellow	Yellow	Yellow	Yellow	Yellow	Green	Yellow	Yellow	Yellow	Yellow	Yellow	Yellow	Yellow	Yellow	Yellow
**IL-1ra (pg/ml)**	Yellow	Yellow	Yellow	Yellow	Yellow	Yellow	Yellow	Yellow	Green	Yellow	Yellow	Yellow	Yellow	Yellow	Yellow	Yellow	Yellow
**CHI3L1 (ng/ml)**	Yellow	Yellow	Yellow	Yellow	Yellow	Yellow	Yellow	Yellow	Yellow	Green	Yellow	Yellow	Yellow	Yellow	Yellow	Yellow	Yellow
**suPAR (ng/ml)**	Yellow	Yellow	Yellow	Yellow	Yellow	Yellow	Yellow	Yellow	Yellow	Yellow	Green	Yellow	Yellow	Yellow	Yellow	Yellow	Yellow
**IL-10 (pg/ml)**	Yellow	Yellow	Yellow	Yellow	Yellow	Yellow	Yellow	Yellow	Yellow	Yellow	Yellow	Red	Yellow	Yellow	Yellow	Yellow	Yellow
**IL-8 (pg/ml)**	Yellow	Yellow	Yellow	Yellow	Yellow	Yellow	Yellow	Yellow	Yellow	Yellow	Yellow	Yellow	Red	Yellow	Yellow	Yellow	Yellow
**IP-10 (pg/ml)**	Yellow	Yellow	Yellow	Yellow	Yellow	Yellow	Yellow	Yellow	Yellow	Yellow	Yellow	Yellow	Yellow	Red	Yellow	Yellow	Yellow
**ANG-1 (pg/ml)**	Yellow	Yellow	Yellow	Yellow	Yellow	Yellow	Yellow	Yellow	Yellow	Yellow	Yellow	Yellow	Yellow	Yellow	Red	Yellow	Yellow
**Glucose (mmol/l)**	Yellow	Yellow	Yellow	Yellow	Yellow	Yellow	Yellow	Yellow	Yellow	Yellow	Yellow	Yellow	Yellow	Yellow	Yellow	Red	Yellow
**Lactate (mmol/l)**	Yellow	Yellow	Yellow	Yellow	Yellow	Yellow	Yellow	Yellow	Yellow	Yellow	Yellow	Yellow	Yellow	Yellow	Yellow	Yellow	Red
**AUC (optimism adjusted)**	0.93	0.93	0.91	0.92	0.92	0.91	0.91	0.91	0.90	0.90	0.91	0.91	0.91	0.91	0.91	0.91	0.91

Green indicates variable was selected, red indicates variable was eliminated, yellow indicates variable was not entered into the model. Model performance is quantified in the derivation cohort using the optimism adjusted weighted AUC.

4. **Table S4: Variable selection and performance of the *clinical-biomarker models* when each biomarker was included in turn alongside the candidate clinical predictors and SpO_2.**

	sTREM1	CRP	PCT	ANG-2	sTNF-R1	sFLT-1	Hb	IL-6	IL-1ra	CHI3L1	suPAR	IL-10	IL-8	IP-10	ANG-1	Glucose	Lactate
**Age (months)**	Red	Red	Red	Red	Red	Red	Red	Red	Red	Red	Red	Red	Red	Red	Red	Red	Red
**MUAC (mm)**	Red	Red	Red	Red	Red	Red	Red	Red	Red	Red	Red	Red	Red	Red	Red	Red	Red
**Heart rate (bpm)**	Green	Green	Green	Green	Green	Green	Green	Green	Green	Green	Green	Green	Green	Green	Green	Green	Green
**Respiratory rate (bpm)**	Green	Green	Green	Green	Green	Green	Green	Green	Green	Green	Green	Green	Green	Green	Green	Green	Green
**Temperature (°C)**	Red	Red	Red	Red	Red	Red	Red	Red	Red	Red	Red	Red	Red	Red	Red	Red	Red
**Altered mental state**	Red	Red	Red	Red	Red	Red	Red	Red	Red	Red	Red	Red	Red	Red	Red	Red	Red
**Prolonged CRT**	Red	Green	Green	Green	Green	Red	Red	Red	Red	Red	Red	Red	Red	Red	Red	Red	Red
**Recent hospitalisation**	Red	Red	Red	Red	Red	Red	Red	Red	Red	Red	Red	Red	Red	Red	Red	Red	Red
**Prostration**	Green	Green	Green	Green	Green	Green	Green	Green	Green	Green	Green	Green	Green	Green	Green	Green	Green
**Intractable vomiting**	Green	Green	Green	Green	Green	Green	Green	Green	Green	Green	Green	Green	Green	Green	Green	Green	Green
**Convulsions**	Red	Red	Red	Red	Red	Red	Green	Green	Green	Green	Green	Green	Green	Green	Green	Green	Green
**SpO_2 (%)**	Green	Green	Green	Green	Green	Green	Green	Green	Green	Green	Green	Green	Green	Green	Green	Green	Green
**sTREM1 (pg/ml)**	Green	Yellow	Yellow	Yellow	Yellow	Yellow	Yellow	Yellow	Yellow	Yellow	Yellow	Yellow	Yellow	Yellow	Yellow	Yellow	Yellow
**CRP (mg/l)**	Yellow	Green	Yellow	Yellow	Yellow	Yellow	Yellow	Yellow	Yellow	Yellow	Yellow	Yellow	Yellow	Yellow	Yellow	Yellow	Yellow
**PCT (ng/ml)**	Yellow	Yellow	Green	Yellow	Yellow	Yellow	Yellow	Yellow	Yellow	Yellow	Yellow	Yellow	Yellow	Yellow	Yellow	Yellow	Yellow
**ANG-2 (pg/ml)**	Yellow	Yellow	Yellow	Green	Yellow	Yellow	Yellow	Yellow	Yellow	Yellow	Yellow	Yellow	Yellow	Yellow	Yellow	Yellow	Yellow
**sTNF-R1 (pg/ml)**	Yellow	Yellow	Yellow	Yellow	Green	Yellow	Yellow	Yellow	Yellow	Yellow	Yellow	Yellow	Yellow	Yellow	Yellow	Yellow	Yellow
**sFLT-1 (pg/ml)**	Yellow	Yellow	Yellow	Yellow	Yellow	Green	Yellow	Yellow	Yellow	Yellow	Yellow	Yellow	Yellow	Yellow	Yellow	Yellow	Yellow
**Hb (g/dl)**	Yellow	Yellow	Yellow	Yellow	Yellow	Yellow	Green	Yellow	Yellow	Yellow	Yellow	Yellow	Yellow	Yellow	Yellow	Yellow	Yellow
**IL-6 (pg/ml)**	Yellow	Yellow	Yellow	Yellow	Yellow	Yellow	Yellow	Green	Yellow	Yellow	Yellow	Yellow	Yellow	Yellow	Yellow	Yellow	Yellow
**IL-1ra (pg/ml)**	Yellow	Yellow	Yellow	Yellow	Yellow	Yellow	Yellow	Yellow	Green	Yellow	Yellow	Yellow	Yellow	Yellow	Yellow	Yellow	Yellow
**CHI3L1 (ng/ml)**	Yellow	Yellow	Yellow	Yellow	Yellow	Yellow	Yellow	Yellow	Yellow	Green	Yellow	Yellow	Yellow	Yellow	Yellow	Yellow	Yellow
**suPAR (ng/ml)**	Yellow	Yellow	Yellow	Yellow	Yellow	Yellow	Yellow	Yellow	Yellow	Yellow	Green	Yellow	Yellow	Yellow	Yellow	Yellow	Yellow
**IL-10 (pg/ml)**	Yellow	Yellow	Yellow	Yellow	Yellow	Yellow	Yellow	Yellow	Yellow	Yellow	Yellow	Red	Yellow	Yellow	Yellow	Yellow	Yellow
**IL-8 (pg/ml)**	Yellow	Yellow	Yellow	Yellow	Yellow	Yellow	Yellow	Yellow	Yellow	Yellow	Yellow	Yellow	Red	Yellow	Yellow	Yellow	Yellow
**IP-10 (pg/ml)**	Yellow	Yellow	Yellow	Yellow	Yellow	Yellow	Yellow	Yellow	Yellow	Yellow	Yellow	Yellow	Yellow	Red	Yellow	Yellow	Yellow
**ANG-1 (pg/ml)**	Yellow	Yellow	Yellow	Yellow	Yellow	Yellow	Yellow	Yellow	Yellow	Yellow	Yellow	Yellow	Yellow	Yellow	Red	Yellow	Yellow
**Glucose (mmol/l)**	Yellow	Yellow	Yellow	Yellow	Yellow	Yellow	Yellow	Yellow	Yellow	Yellow	Yellow	Yellow	Yellow	Yellow	Yellow	Red	Yellow
**Lactate (mmol/l)**	Yellow	Yellow	Yellow	Yellow	Yellow	Yellow	Yellow	Yellow	Yellow	Yellow	Yellow	Yellow	Yellow	Yellow	Yellow	Yellow	Red
**AUC (optimism adjusted)**	0.94	0.94	0.93	0.92	0.92	0.92	0.92	0.92	0.92	0.92	0.91	0.91	0.91	0.91	0.91	0.91	0.91

Green indicates variable was selected, red indicates variable was eliminated, yellow indicates variable was not entered into the model. Model performance is quantified in the derivation cohort using the optimism adjusted weighted AUC.

5. **Table S5: Variable selection and performance of the *clinical-biomarker models* when each biomarker was included in turn alongside the candidate clinical predictors, excluding the northern Viet Nam site.**

	sTREM1	CRP	PCT	ANG-2	sTNF-R1	sFLT-1	Hb	IL-6	IL-1ra	CHI3L1	suPAR	IL-10	IL-8	IP-10	ANG-1	Glucose	Lactate
**Age (months)**	Red	Red	Red	Red	Red	Red	Red	Red	Red	Red	Red	Red	Red	Red	Red	Red	Red
**MUAC (mm)**	Red	Red	Red	Red	Red	Red	Red	Red	Red	Red	Red	Red	Red	Red	Red	Red	Red
**Heart rate (bpm)**	Green	Green	Green	Green	Green	Green	Green	Green	Green	Green	Green	Green	Green	Green	Green	Green	Green
**Respiratory rate (bpm)**	Green	Green	Green	Green	Green	Green	Green	Green	Green	Green	Green	Green	Green	Green	Green	Green	Green
**Temperature (°C)**	Red	Red	Red	Red	Red	Red	Red	Red	Red	Red	Red	Red	Red	Red	Red	Red	Red
**Altered mental state**	Red	Red	Red	Red	Red	Red	Red	Red	Red	Red	Red	Red	Red	Red	Red	Red	Red
**Prolonged CRT**	Red	Red	Red	Red	Red	Red	Red	Red	Red	Red	Red	Red	Red	Red	Red	Red	Red
**Recent hospitalisation**	Red	Red	Red	Red	Red	Red	Red	Red	Red	Red	Red	Red	Red	Red	Red	Red	Red
**Prostration**	Green	Green	Green	Green	Green	Green	Green	Green	Green	Green	Green	Green	Green	Green	Green	Green	Green
**Intractable vomiting**	Green	Green	Green	Green	Green	Green	Green	Green	Green	Green	Green	Green	Green	Green	Green	Green	Green
**Convulsions**	Green	Green	Green	Green	Green	Red	Green	Green	Green	Green	Green	Green	Green	Green	Green	Green	Green
**SpO_2 (%)**	Green	Green	Green	Green	Green	Green	Green	Green	Green	Green	Green	Green	Green	Green	Green	Green	Green
**sTREM1 (pg/ml)**	Green	Yellow	Yellow	Yellow	Yellow	Yellow	Yellow	Yellow	Yellow	Yellow	Yellow	Yellow	Yellow	Yellow	Yellow	Yellow	Yellow
**CRP (mg/l)**	Yellow	Red	Yellow	Yellow	Yellow	Yellow	Yellow	Yellow	Yellow	Yellow	Yellow	Yellow	Yellow	Yellow	Yellow	Yellow	Yellow
**PCT (ng/ml)**	Yellow	Yellow	Green	Yellow	Yellow	Yellow	Yellow	Yellow	Yellow	Yellow	Yellow	Yellow	Yellow	Yellow	Yellow	Yellow	Yellow
**ANG-2 (pg/ml)**	Yellow	Yellow	Yellow	Green	Yellow	Yellow	Yellow	Yellow	Yellow	Yellow	Yellow	Yellow	Yellow	Yellow	Yellow	Yellow	Yellow
**sTNF-R1 (pg/ml)**	Yellow	Yellow	Yellow	Yellow	Green	Yellow	Yellow	Yellow	Yellow	Yellow	Yellow	Yellow	Yellow	Yellow	Yellow	Yellow	Yellow
**sFLT-1 (pg/ml)**	Yellow	Yellow	Yellow	Yellow	Yellow	Green	Yellow	Yellow	Yellow	Yellow	Yellow	Yellow	Yellow	Yellow	Yellow	Yellow	Yellow
**Hb (g/dl)**	Yellow	Yellow	Yellow	Yellow	Yellow	Yellow	Green	Yellow	Yellow	Yellow	Yellow	Yellow	Yellow	Yellow	Yellow	Yellow	Yellow
**IL-6 (pg/ml)**	Yellow	Yellow	Yellow	Yellow	Yellow	Yellow	Yellow	Red	Yellow	Yellow	Yellow	Yellow	Yellow	Yellow	Yellow	Yellow	Yellow
**IL-1ra (pg/ml)**	Yellow	Yellow	Yellow	Yellow	Yellow	Yellow	Yellow	Yellow	Red	Yellow	Yellow	Yellow	Yellow	Yellow	Yellow	Yellow	Yellow
**CHI3L1 (ng/ml)**	Yellow	Yellow	Yellow	Yellow	Yellow	Yellow	Yellow	Yellow	Yellow	Red	Yellow	Yellow	Yellow	Yellow	Yellow	Yellow	Yellow
**suPAR (ng/ml)**	Yellow	Yellow	Yellow	Yellow	Yellow	Yellow	Yellow	Yellow	Yellow	Yellow	Green	Yellow	Yellow	Yellow	Yellow	Yellow	Yellow
**IL-10 (pg/ml)**	Yellow	Yellow	Yellow	Yellow	Yellow	Yellow	Yellow	Yellow	Yellow	Yellow	Yellow	Red	Yellow	Yellow	Yellow	Yellow	Yellow
**IL-8 (pg/ml)**	Yellow	Yellow	Yellow	Yellow	Yellow	Yellow	Yellow	Yellow	Yellow	Yellow	Yellow	Yellow	Red	Yellow	Yellow	Yellow	Yellow
**IP-10 (pg/ml)**	Yellow	Yellow	Yellow	Yellow	Yellow	Yellow	Yellow	Yellow	Yellow	Yellow	Yellow	Yellow	Yellow	Red	Yellow	Yellow	Yellow
**ANG-1 (pg/ml)**	Yellow	Yellow	Yellow	Yellow	Yellow	Yellow	Yellow	Yellow	Yellow	Yellow	Yellow	Yellow	Yellow	Yellow	Red	Yellow	Yellow
**Glucose (mmol/l)**	Yellow	Yellow	Yellow	Yellow	Yellow	Yellow	Yellow	Yellow	Yellow	Yellow	Yellow	Yellow	Yellow	Yellow	Yellow	Red	Yellow
**Lactate (mmol/l)**	Yellow	Yellow	Yellow	Yellow	Yellow	Yellow	Yellow	Yellow	Yellow	Yellow	Yellow	Yellow	Yellow	Yellow	Yellow	Yellow	Red
**AUC (optimism adjusted)**	0.98	0.97	0.97	0.98	0.97	0.97	0.98	0.97	0.97	0.97	0.97	0.97	0.97	0.97	0.97	0.97	0.97

Green indicates variable was selected, red indicates variable was eliminated, yellow indicates variable was not entered into the model. Model performance is quantified in the derivation cohort using the optimism adjusted weighted AUC.

6. Tables S6: Logistic regression equations for the four clinical prediction models.

Instructions to use these equations:

1. Input the value for each variable.
2. Compute the linear predictor (LP), which is the value of the right-hand side of the equation.
3. Convert the LP to a probability using the logistic function: $p = 1 / (1 + e^{(-LP)})$
4. The result p is the predicted probability

Clinical model

Variable	Coefficient (β)	Odds Ratio (95% CI)
Constant	-13.8215	-
Intractable vomiting	1.1946	3.30 (1.78-6.13)
Prostration	2.1689	8.75 (4.26-17.97)
Heart rate	0.0322	1.03 (1.02-1.05)
Respiratory rate	0.0624	1.06 (1.04-1.09)
Prolonged capillary refill time	1.0570	2.88 (1.26-6.58)
Altered mental state	1.9074	6.74 (2.06-22.07)

Logistic Regression Equation: log(p / (1 - p)) = -13.8215 + (1.1946*vomit) + (2.1689*prostration) + (0.0322*heart_rate) + (0.0624*respiratory_rate) + (1.0570*cap_refill_time) + (1.9074*mental_state)

Pulse oximetry model

Variable	Coefficient (β)	Odds Ratio (95% CI)
Constant	1.9561	-
Convulsions	1.0210	2.78 (1.23-6.27)
Intractable vomiting	1.2767	3.58 (2.02-6.37)
Prostration	2.1623	8.69 (4.45-16.97)
Heart rate	0.0268	1.03 (1.01-1.04)
Respiratory rate	0.0624	1.06 (1.04-1.09)
SpO_2	-0.1541	0.86 (0.80-0.92)

Logistic Regression Equation: log(p / (1 - p)) = 1.9561 + (1.0210*convulsion) + (1.2767*vomit) + (2.1623*prostration) + (0.0268*heart_rate) + (0.0624*respiratory_rate) – (0.1541*SpO_2)

sTREM1 model

Variable	Coefficient (β)	Odds Ratio (95% CI)
Constant	-14.5167	-
Intractable vomiting	1.2290	3.42 (1.90-6.16)
Prostration	1.9687	7.16 (3.66-14.02)
Heart rate	0.0342	1.03 (1.02-1.05)
Respiratory rate	0.0540	1.06 (1.03-1.09)
Altered mental state	2.0153	7.50 (2.30-24.43)
sTREM1	0.0030	1.00 (1.00-1.00)

Logistic Regression Equation: log(p / (1 - p)) = -14.5167 + (1.2290*vomit) + (1.9687*prostration) + (0.0342*heart_rate) + (0.0540*respiratory_rate) + (2.0153*mental_state) + (0.0030*sTREM1)

Combined model

Variable	Coefficient (β)	Odds Ratio (95% CI)
Constant	0.5857	-
Prostration	1.7803	5.93 (2.90-12.14)
Intractable vomiting	1.3179	3.74 (2.07-6.74)
Heart rate	0.0290	1.03 (1.02-1.04)
Respiratory rate	0.0504	1.05 (1.02-1.08)
sTREM1	0.0029	1.00 (1.00-1.00)
SpO_2	-0.1454	0.86 (0.80-0.93)

Logistic Regression Equation: $\log(p / (1 - p)) = 0.5857 + (1.7803*prostration) + (1.3179*vomit) + (0.0290*heart\_rate) + (0.0504*respiratory\_rate) + (0.0029*sTREM1) – (0.1454*SpO_2)$

7. Table S7: Categorical outcome scale.

Category	Definition
**I**	Not admitted to any health facility between enrolment and D28 AND recovered by D28
**II**	Admission to any health facility for ≤ 2 nights between enrolment and D28 OR symptoms not resolved by D28
**III**	Admission to any health facility for > 2 nights between enrolment and D28 OR death or organ support between D2 and D28
**IV**	Death or organ support ≤ 2 days after enrolment*

*Category IV equivalent to the primary outcome. Outcome categories were determined in descending order of severity and were mutually exclusive, i.e. if a participant met the criteria for Category IV, this would be their classification.

8. Table S8: Characteristics of participants who developed severe disease, stratified by whether they were identified by each prediction model (derivation and validation cohorts pooled).

Characteristic	Severe patients N = 133[1]	Clinical model		Pulse oximetry model		sTREM1 model		Combined model	
		Identified N = 102[1]	Missed N = 31[1]	Identified N = 107[1]	Missed N = 26[1]	Identified N = 111[1]	Missed N = 22[1]	Identified N = 110[1]	Missed N = 23[1]
**Demographics and background**									
Age (months)	4.9 (2.6, 17.3)	4.5 (2.5, 11.2)	25.3 (4.9, 43.9)	4.5 (2.1, 12.3)	30.2 (6.3, 48.1)	4.5 (2.1, 12.7)	29.0 (6.3, 49.7)	4.5 (2.5, 12.7)	27.7 (4.9, 49.7)
Male sex	45 (34%)	30 (29%)	15 (48%)	31 (29%)	14 (54%)	34 (31%)	11 (50%)	33 (30%)	12 (52%)
**Anthropometrics**									
Wasted (WHZ < -2)*	34 (26%)	30 (29%)	4 (13%)	29 (27%)	5 (19%)	30 (27%)	4 (18%)	30 (28%)	4 (17%)
Stunted (HAZ < -2)	35 (26%)	27 (26%)	8 (26%)	32 (30%)	3 (12%)	32 (29%)	3 (14%)	32 (29%)	3 (13%)
MUAC-for-age z-score[a]	-0.5 (-1.5, 0.6)	-0.9 (-1.7, 0.3)	-0.2 (-0.8, 0.9)	-0.7 (-1.6, 0.4)	-0.2 (-0.9, 0.9)	-0.7 (-1.6, 0.4)	0.0 (-0.8, 0.9)	-0.7 (-1.6, 0.5)	-0.2 (-0.8, 0.9)
**Illness characteristics**									
Duration of illness (days)	3.0 (2.0, 5.0)	4.0 (3.0, 5.0)	2.0 (1.0, 3.0)	4.0 (3.0, 5.0)	2.0 (1.0, 4.0)	4.0 (3.0, 5.0)	2.0 (1.0, 3.0)	4.0 (3.0, 5.0)	2.0 (1.0, 4.0)
URTI	39 (29%)	29 (28%)	10 (32%)	33 (31%)	6 (23%)	33 (30%)	6 (27%)	34 (31%)	5 (22%)
LRTI	86 (65%)	76 (75%)	10 (32%)	80 (75%)	6 (23%)	80 (72%)	6 (27%)	81 (74%)	5 (22%)
Diarrhoeal	15 (11%)	12 (12%)	3 (9.7%)	12 (11%)	3 (12%)	12 (11%)	3 (14%)	12 (11%)	3 (13%)
Neurological	14 (11%)	12 (12%)	2 (6.5%)	12 (11%)	2 (7.7%)	12 (11%)	2 (9.1%)	12 (11%)	2 (8.7%)
No focus	8 (6.0%)	7 (6.9%)	1 (3.2%)	8 (7.5%)	0 (0%)	8 (7.2%)	0 (0%)	8 (7.3%)	0 (0%)
**WHO danger signs**									
Any WHO danger sign*	95 (72%)	76 (75%)	19 (61%)	80 (75%)	15 (58%)	82 (75%)	13 (59%)	81 (74%)	14 (61%)
Prostration	48 (36%)	47 (46%)	1 (3.2%)	47 (44%)	1 (3.8%)	48 (43%)	0 (0%)	47 (43%)	1 (4.3%)
Intractable vomiting	42 (32%)	32 (31%)	10 (32%)	33 (31%)	9 (35%)	33 (30%)	9 (41%)	33 (30%)	9 (39%)
*Convulsions**	14 (11%)	12 (12%)	2 (6.5%)	13 (12%)	1 (3.8%)	12 (11%)	2 (9.1%)	13 (12%)	1 (4.3%)
*Lethargy**	61 (46%)	52 (51%)	9 (29%)	55 (52%)	6 (23%)	58 (53%)	3 (14%)	56 (51%)	5 (22%)
**Vital signs**									
Heart rate (bpm)									
1 to 12 months (bpm)	171.5 (157.0, 186.0)	172.0 (160.0, 189.0)	150.0 (146.0, 175.0)	172.0 (158.5, 189.5)	155.0 (143.0, 172.0)	172.0 (159.0, 189.0)	148.0 (140.0, 160.0)	172.0 (158.5, 189.5)	155.0 (143.0, 172.0)
12 to 60 months (bpm)	160.0 (140.0, 177.0)	172.0 (160.0, 192.0)	144.0 (130.5, 154.5)	171.0 (156.0, 190.0)	141.0 (129.0, 158.0)	170.0 (158.0, 190.0)	141.0 (130.5, 150.0)	170.5 (158.0, 190.0)	136.0 (127.0, 150.0)
Respiratory rate (bpm)									
1 to 12 months (bpm)	59.5 (48.0, 66.0)	60.0 (50.0, 68.0)	42.0 (35.0, 50.0)	60.0 (50.0, 67.5)	41.0 (37.5, 44.0)	60.0 (50.0, 67.0)	40.0 (35.0, 42.0)	60.0 (50.0, 67.5)	41.0 (37.5, 44.0)
1 to 12 months (bpm)	39.0 (32.0, 55.0)	55.0 (40.0, 67.0)	32.0 (28.5, 37.5)	54.0 (39.0, 67.0)	32.0 (28.0, 38.0)	50.0 (39.0, 65.0)	31.5 (28.0, 37.5)	50.0 (39.0, 65.0)	31.0 (28.0, 37.0)
Oxygen saturation (%)*	97.0 (95.0, 98.0)	96.0 (93.0, 98.0)	98.0 (97.0, 98.0)	96.0 (92.0, 98.0)	98.0 (97.0, 99.0)	96.0 (93.0, 98.0)	98.0 (97.0, 98.0)	96.0 (93.0, 98.0)	98.0 (97.0, 99.0)
Axillary temperature (°C)	37.6 (36.9, 38.3)	37.6 (37.0, 38.3)	37.5 (36.7, 38.4)	37.6 (36.9, 38.3)	37.7 (37.0, 38.4)	37.6 (36.9, 38.3)	37.5 (36.8, 38.3)	37.6 (36.9, 38.3)	37.5 (36.8, 38.3)
CRT > 2 seconds	29 (22%)	28 (27%)	1 (3.2%)	28 (26%)	1 (3.8%)	28 (25%)	1 (4.5%)	28 (25%)	1 (4.3%)
Not alert[b]	19 (14%)	19 (19%)	0 (0%)	18 (17%)	1 (3.8%)	19 (17%)	0 (0%)	18 (16%)	1 (4.3%)
**Endothelial activation markers**									
ANG-1 (pg/ml)*	5,746.0 (3,426.0, 10,543.0)	6,345.5 (3,658.0, 11,662.0)	4,562.0 (3,155.0, 6,225.0)	6,043.0 (3,610.0, 10,369.0)	5,178.5 (3,155.0, 11,270.0)	6,049.0 (3,698.0, 10,894.0)	3,760.5 (2,977.0, 6,225.0)	5,998.5 (3,614.0, 10,456.0)	4,899.0 (2,977.0, 11,270.0)
ANG-2 (pg/ml)*	2,532.0 (1,435.0, 4,361.0)	3,262.0 (1,763.5, 5,185.0)	1,546.0 (1,044.0, 2,776.0)	3,249.0 (1,784.0, 5,168.0)	1,514.0 (1,011.0, 2,272.0)	3,275.0 (1,784.0, 5,168.0)	1,410.5 (982.0, 1,905.0)	3,244.5 (1,763.5, 5,148.5)	1,494.0 (982.0, 2,272.0)
sFLT-1 (pg/ml)*	258.0 (202.0, 371.0)	274.0 (222.0, 462.0)	211.0 (170.0, 273.0)	274.0 (218.0, 459.0)	210.5 (165.0, 268.0)	274.0 (218.0, 459.0)	208.0 (165.0, 242.0)	268.5 (212.5, 452.0)	211.0 (165.0, 273.0)

Characteristic	Severe patients N = 133[1]	Clinical model		Pulse oximetry model		sTREM1 model		Combined model	
		Identified N = 102[1]	Missed N = 31[1]	Identified N = 107[1]	Missed N = 26[1]	Identified N = 111[1]	Missed N = 22[1]	Identified N = 110[1]	Missed N = 23[1]
**Immune activation markers**									
CHI3L1 (ng/ml)*	37.8 (24.0, 66.4)	38.9 (25.4, 80.1)	29.7 (22.4, 49.5)	38.9 (24.7, 78.1)	29.9 (23.6, 49.0)	38.9 (25.5, 75.3)	29.2 (15.6, 48.5)	38.9 (24.0, 76.2)	29.7 (21.4, 49.0)
CRP (mg/l)*	25.8 (5.0, 105.3)	22.7 (3.1, 80.5)	42.9 (9.3, 166.8)	21.8 (3.6, 78.6)	89.0 (16.2, 200.7)	23.5 (3.2, 80.0)	57.8 (9.8, 166.8)	23.6 (3.8, 80.5)	42.9 (9.3, 166.8)
IL-1ra (pg/ml)*	2,829.0 (1,211.0, 9,799.0)	3,509.5 (1,372.0, 11,423.0)	1,756.0 (779.0, 7,223.0)	3,247.0 (1,414.0, 10,537.0)	1,701.0 (904.0, 7,223.0)	3,228.0 (1,235.0, 10,537.0)	1,803.0 (904.0, 7,442.0)	3,237.5 (1,282.5, 10,332.0)	1,756.0 (779.0, 7,442.0)
IL-6 (pg/ml)*	51.8 (11.3, 211.0)	51.3 (11.8, 195.0)	60.7 (9.5, 220.0)	50.7 (11.7, 179.0)	73.5 (11.3, 220.0)	51.8 (11.9, 215.0)	59.4 (9.5, 176.0)	51.3 (11.8, 213.0)	73.2 (9.5, 183.0)
IL-8 (pg/ml)*	28.7 (14.2, 85.8)	36.9 (18.2, 113.0)	15.7 (6.6, 27.4)	35.7 (18.1, 102.0)	13.0 (6.6, 19.9)	35.7 (17.6, 102.0)	13.0 (6.6, 18.6)	35.6 (17.5, 106.0)	15.0 (6.6, 19.9)
IL-10 (pg/ml)*	27.5 (14.0, 70.0)	33.6 (16.9, 78.3)	20.3 (8.0, 54.8)	33.6 (17.9, 71.3)	12.7 (7.1, 54.8)	33.5 (16.5, 72.4)	16.0 (7.1, 54.8)	33.6 (17.1, 71.9)	14.3 (7.1, 54.8)
IP-10 (pg/ml)*	591.0 (234.0, 1,258.0)	678.0 (256.0, 1,209.5)	350.0 (143.0, 1,457.0)	652.0 (258.0, 1,258.0)	316.5 (109.0, 1,065.0)	630.0 (248.0, 1,137.0)	347.5 (109.0, 3,004.0)	660.0 (256.0, 1,259.0)	288.0 (82.4, 927.0)
PCT (ng/ml)*	0.8 (0.4, 3.7)	1.0 (0.5, 5.1)	0.5 (0.2, 1.5)	1.0 (0.5, 4.4)	0.5 (0.2, 1.0)	1.0 (0.5, 4.9)	0.3 (0.2, 1.0)	1.0 (0.5, 5.1)	0.4 (0.2, 1.0)
sTNF-R1 (pg/ml)*	2,121.0 (1,549.0, 3,230.0)	2,245.5 (1,590.5, 3,790.0)	1,752.0 (1,356.0, 2,266.0)	2,267.0 (1,585.0, 3,603.0)	1,746.0 (1,356.0, 1,936.0)	2,266.0 (1,596.0, 3,548.0)	1,580.5 (1,356.0, 1,839.0)	2,245.0 (1,581.0, 3,575.5)	1,740.0 (1,356.0, 1,936.0)
sTREM1 (pg/ml)*	376.0 (273.0, 564.0)	420.5 (286.0, 619.0)	344.0 (205.0, 421.0)	415.0 (282.0, 615.0)	344.0 (214.0, 421.0)	420.0 (301.0, 623.0)	225.0 (198.0, 348.0)	417.5 (286.0, 624.0)	261.0 (205.0, 416.0)
suPAR (ng/ml)*	5.4 (4.1, 7.7)	5.7 (4.4, 8.0)	4.3 (2.7, 5.6)	5.6 (4.4, 8.0)	3.6 (2.6, 5.8)	5.6 (4.4, 8.1)	3.6 (2.7, 5.0)	5.6 (4.4, 8.1)	3.2 (2.6, 4.8)
**Other laboratory markers**									
Lactate (mmol/l)*	1.4 (0.8, 2.1)	1.4 (0.9, 2.4)	1.0 (0.6, 1.7)	1.4 (0.9, 2.5)	0.9 (0.6, 1.7)	1.4 (0.9, 2.5)	0.7 (0.6, 1.6)	1.4 (0.9, 2.4)	0.8 (0.6, 1.9)
Glucose (mmol/l)*	6.0 (5.1, 7.1)	6.1 (5.1, 7.3)	5.7 (5.1, 6.8)	6.1 (5.1, 7.3)	5.7 (5.1, 6.8)	6.1 (5.2, 7.3)	5.7 (5.0, 6.8)	6.1 (5.1, 7.2)	5.7 (5.1, 6.8)
Hb (g/dL)*	10.8 (9.5, 12.1)	10.3 (9.3, 11.8)	11.8 (10.9, 12.2)	10.4 (9.3, 11.9)	11.6 (10.6, 12.5)	10.4 (9.3, 11.9)	12.0 (10.9, 12.5)	10.4 (9.3, 11.9)	11.8 (10.6, 12.5)
**Prognostication**									
Time to event (hours)*	6.0 (1.0, 21.0)	3.0 (1.0, 20.5)	9.0 (4.0, 21.0)	4.0 (1.0, 24.0)	9.0 (4.0, 17.0)	4.0 (1.0, 18.0)	10.5 (6.0, 21.0)	4.0 (1.0, 23.5)	10.0 (4.0, 19.0)

[1]Median (Q1, Q3); n (%)

[a]Calculated in children aged 3-60 months; [b]Assessed using the Alert Voice Pain Unresponsive (AVPU) scale. CRT = capillary refill time; LRTI = lower respiratory tract infection; URTI = upper respiratory tract infection.

*Missing data: wasted, n = 1; MUAC-for-age z-score, n = 41; WHO danger sign, n = 1; convulsions, n = 1; lethargy, n = 1, oxygen saturation, n = 60; ANG-1, ANG-2, sFlt-1, CRP, IL-1ra, IL-6, IL-8, IL-10, IP-10, PCT, sTNF-R1, sTREM1, n = 6; CHI3L1, lactate, glucose, n = 7; suPAR, n = 8; Hb, n = 13; time to severe disease, n = 6.

9. Table S9: Variation in predicted cost-effectiveness with increasing referral costs.

Comparator: WHO danger signs

All models were predicted to be cost-effective irrespective of referral cost (all models had better sensitivity and specificity).

Comparator: clinical model

The *pulse oximetry model* and the *combined model* had better specificity compared to the *clinical model.* As a result, cost-effectiveness was predicted to improve as referral costs increased. For the *sTREM1 model*, the table indicates the maximum referral cost per patient at which it was predicted to remain cost-effective compared to the *clinical model*, using the two cost-effectiveness thresholds (CETs).

Maximum referral cost per patient at which models remain cost-effective

**Model**	**Cost-effective up to maximum referral cost vs. *clinical model* (USD)**	
	**CET = $459/DALY averted**	**CET = $2,551/DALY averted**
sTREM1 model	$593	$5,217

Comparator: pulse oximetry model

The *pulse oximetry model* was predicted to be cost-effective compared to the *clinical model* with an incremental cost-effectiveness ratio (ICER) of $26/DALY averted, and due to its better specificity cost-effectiveness was predicted to improve as referral costs increased. Neither the *sTREM1* nor *combined models*, were predicted to be cost-effective compared to the *pulse oximetry model* using either of the two CETs.

10. Table S10: Methodology for collection of clinical parameters.

Variable	Methodology
Respiratory rate	Manual count for 60 seconds using clicker counter and timer
Heart rate	Massimo Rad-5v pulse oximeter with paediatric and neonatal probes
Oxygen saturation	Massimo Rad-5v pulse oximeter with paediatric and neonatal probes
Axillary temperature	Digital thermometer: operating range 32.0-42.9°C; accuracy ± 0.1°C
Capillary refill time	Pressure applied to sternum for 5 seconds
Length / Height	*Médecins Sans Frontières* height and length board
Weight	Seca 877 scale with mother-and-child function; accuracy ± 50g
Mid-upper arm circumference	*Médecins Sans Frontières* traffic light MUAC tape
Mental state	Alert Voice Pain Unresponsive (AVPU) scale
WHO danger signs	WHO IMCI Distance Learning course: https://iris.who.int/handle/10665/104772

11. **Table S11: Existing evidence supporting selection of endothelial and immune activation markers.**

Biomarker	Overview of supportive data
**Endothelial activation**	
**ANG-1 and -2**	Supportive data from Asia/SSA/Europe in children/adults, that increases in ANG-2, decreases in ANG-1, and/or the ANG-2:1 ratio predicts mortality in pneumonia, malaria, SBI, and all-cause febrile illnesses, and supplemental oxygen requirement in children with pneumonia in Asia.
**sFLT-1**	Supportive data from SSA that increases in sFLT-1 predict mortality in children hospitalised with pneumonia, severe malaria, and all-cause febrile illnesses, and adults with all-cause febrile illnesses.
**Immune activation**	
**CHI3L1**	Supportive data from SSA that increases in CHI3L1 predict mortality in children hospitalised with pneumonia and all-cause febrile illnesses, and adults with all-cause febrile illnesses.
**CRP**	Although there is limited supportive evidence for the use of CRP as a prognostic marker for disease severity, as it is the most widely studied biomarker in the region, and numerous point-of-care tests already exist, further evaluation is warranted.
**IL-1ra**	Supportive data that increases in IL-1ra are associated with severity in children with meningococcal disease, adults with SARS-CoV-2 infection, and predict need for longer antibiotic duration in children with febrile lower respiratory tract infections.
**IL-6**	Supportive data from India that increases in IL-6 are predictive of mortality in children with dengue; in Switzerland, supportive data that increases in IL-6 predict need for longer antibiotic duration in children with febrile lower respiratory tract infection, and disease severity in adults with SARS-CoV-2 infection.
**IL-8**	Supportive data from India that increases in IL-8 predict mortality in children with dengue; in Mozambique, IL-8 predicted mortality in children with pneumonia; in the UK, supportive data that increases in IL-8 predict disease severity in children with meningococcal disease.
**IL-10**	Supportive data from India that increases in IL-10 predict of mortality in children with dengue.
**IP-10**	Supportive data from Uganda that increases in IP-10 predict mortality in children hospitalised with severe malaria.
**PCT**	Supportive evidence that increases in PCT predict severe illness in hospitalised children with suspected bacterial infections or meningococcal disease.
**sTNF-R1**	Supportive data from SSA that increases in sTNF-R1 predict mortality in children hospitalised with pneumonia and all-cause febrile illnesses, and adults with all-cause febrile illnesses.
**sTREM1**	Supportive data from SSA that increases in sTREM1 predict mortality in children hospitalised with pneumonia, severe malaria, and all-cause febrile illnesses, and adults with all-cause febrile illnesses; in Asia, increased sTREM1 predicted length of stay in infant febrile illness and in-hospital mortality in adults hospitalised with infection and children hospitalised with pneumonia.
**suPAR**	Supportive data from Uganda that increases in suPAR predict mortality in children with malaria; In Europe, elevated suPAR concentrations predicted length of stay in children with pneumonia and mortality in adults hospitalised with sepsis.

12. Table S12: Laboratory procedures for biomarker quantification.

Host biomarker concentrations were quantified in EDTA-plasma using the Simple Plex Ella microfluidic platform (ProteinSimple, San Jose, CA, USA) and suPARnostic ELISA (ViroGates, Denmark), or fluoride-oxalate-plasma using LACT2 (Roche Diagnostics, Germany) and GLUC3 (Roche Diagnostics, Germany), according to the manufacturers' protocols. All biomarkers were quantified at the MORU laboratories in Bangkok, Thailand, apart from Indonesian samples, which were quantified at the INA-RESPOND laboratories in Jakarta, Indonesia, with consumables and on-site support provided by the visiting MORU laboratory team.

For the Ella platform, plasma samples were diluted 1:2 (ANG-2, IL-6, IL-8, IL-10, PCT, sTREM1, sFLT-1), 1:10 (ANG-1, CHI3L1, IL-1ra, IP-10, sTNF-R1), or 1:5000 (CRP) in reagent diluent. Analyte concentrations outside the dynamic range of the assay using the initial dilutions were prepared at higher or lower dilutions depending on the raw data relative fluorescent units (RFUs). For suPAR, no dilutions to plasma were performed prior to adding the samples to the pre-coated ELISA plate. Any samples with concentrations above or below the assays' limit of detection were assigned a value of one-third of the highest or lowest limit, respectively, of the limits of quantification. The table below details the proportion of samples assigned a value.

	**Dynamic Range** (pg/mL)	**Samples outside dynamic range**	**Proportion assigned a value**
ANG-1	6.18 - 23,560	0	-
ANG-2	9.91 - 15,124	0	-
CHI3L1	6.68 - 25,500	2	2/3,312 (0.06%)
CRP	32.8 - 50,000	5	5/3,309 (0.15%)
IL-1ra	7.37 - 4,500	1	1/3,313 (0.03%)
IL-6	0.28 - 2,652	4	4/3,314 (0.12%)
IL-8	0.19 - 1,804	2	2/3,314 (0.06%)
IL-10	0.58 - 2,212	2	2/3,314 (0.06%)
IP-10	0.6 - 920	0	-
PCT	1.58 - 15,100	0	-
sFLT-1	3.05 - 4,650	0	-
sTNF-R1	0.89 - 3,390	0	-
sTREM1	4.2 - 40,000	1	1/3,314 (0.03%)
suPAR	400 - 16,000*	15	-

*Samples outside the dynamic range (>16,000 pg/mL) for suPAR could be estimated correctly up to 25,000 pg/mL and thus new values were not assigned.

13. Table S13: Derivation of site-specific outpatient weights.

	Bangladesh	Cambodia	Laos 1	Laos 2	Viet Nam 1	Viet Nam 2	Total
**Screening week data**							
Number of screening weeks	3	4	2	1	2	-	-
Patients screened (A)	1,556	1,872	204	39	899	-	-
Patients eligible (B)	320	346	71	4	180	-	-
Proportion eligible (B/A = C)	0.21	0.19	0.35	0.10	0.20	0.20	-
**Routine hospital data**							
Outpatient attendance during study (D)	28,613	59,853	5,403	1,278	48,296	36,854	-
Estimated number eligible (C*D = E)	5,867	11,073	1,891	128	9,660	7,371	-
**Study data**							
Number of outpatients recruited (F)	175	183	87	29	147	147	-
Outpatient weighting (1 : E/F)	1 : 34	1 : 64	1 : 22	1 : 5	1 : 66	1 : 50	-
**Outcomes**							
Frequency	39/546	36/816	0/205	0/77	32/938	26/611	
Unweighted prevalence (95% CI)	7.1% (5.1-9.6)	4.4% (3.1-6.1)	-	-	3.4% (2.3-4.8)	4.3% (2.8-6.2)	**3.9% (3.3-4.7)**
Weighted prevalence (95% CI)	0.64% (0.45-0.90)	0.30% (0.20-0.42)	-	-	0.31% (0.21-0.45)	0.39% (0.26-0.59)	**0.34% (0.28-0.41)**

Due to high numbers of outpatients, consecutive enrolment of outpatients was not feasible, and recruitment was stratified by admission status, with consecutive screening of inpatients, whilst outpatients were randomly selected for screening. To account for this, the outpatient strata was weighted in the analyses. For one week every 4-6 months, outpatient recruitment was paused and consecutive attendances screened for eligibility. These screening week data were triangulated with routinely collected hospital attendance data to estimate the total number of eligible outpatients presenting to each site during the recruitment period, to determine the weights to be used in the analyses.

Accordingly, screening weeks were planned at each site where outpatient screening was randomised. At study inception outpatient screening was randomised at all sites. Following the start of the Covid-19 pandemic, attendance rates at the site in Indonesia decreased substantially, such that consecutive outpatient screening became possible. The switch to consecutive screening in Indonesia occurred on 10/04/2021 (three weeks after site initiation), covering 90.4% (66/73) of outpatient recruitment at that site. As consecutive screening was used for the majority of outpatient recruitment in Indonesia, no weighting was necessary. However, to align with the methodology used at the other sites, where the estimated number of eligible outpatients presenting to the study site during the recruitment period (E) assumed no refusals, the Indonesian outpatient data were weighted by the observed outpatient refusal rate (62/135; 46%), to provide a weighting of 1:2 for the Indonesian outpatient data.

The second Viet Nam site joined the study on 8 December 2021 in order to boost recruitment, which had been delayed by the Covid-19 pandemic (appendix p15-16). Due to limited remaining resources at this stage of the study, it was not possible to recruit outpatients at this site. Therefore, outpatient data from the first Viet Nam site are used as a proxy. Weighting of these data for the second Viet Nam site was performed using the ratio of inpatients recruited at each Vietnamese site (612:802) and the total number of outpatient attendances at the first Viet Nam site (48,296), to estimate the number of outpatients that would have presented to the second Viet Nam site: 48,296 * (612/802) = 36,854. Assuming the same proportion (0.20) of eligible outpatients at both Viet Nam sites, the number of eligible outpatients presenting to the second Viet Nam site was estimated as: 36,854 * 0.20 = 7,371. This number was then used to determine the weighting to apply to the outpatient data from the first Viet Nam site, as a proxy for outpatient data at the second Viet Nam site: 7,371/147 = 50. Key assumptions underlying this are that the ratio of inpatients to outpatients, proportion of eligible outpatients, and profile of outpatients are similar at both Vietnamese sites.

14. Table S14: Parameters for cost-effectiveness analyses.

Parameter	Value	Source	Notes
Average population age	1 year	Primary data	16.8 months, rounded to 1 year
Life expectancy at population age	75.21 years	UN Population Prospects 2024	Life expectancy at age x - Bangladesh
Percentage of patients that will develop severe symptoms	0.3%	Primary data	133/3405 patients (weighted)
Mortality if severe and referred	16.54%	Primary data	22/133 patients
Mortality if severe and not referred	100%	Assumed	
SpO_2 cost per patient	$0.45	Chew et al. 2022	$275 purchase cost and $55 maintenance cost. Assumed two patients per day with a useful lifespan of one year
sTREM1 cost per patient	$3.87	Calarco et al. 2023	Assume cost same as CRP. Used the median cost of analyser ($1000) and consumables ($2.50). Assume used by 2 patients per day with a useful lifespan of one year
Outpatient appointment cost per patient for initial assessment	$2.80	WHO-CHOICE	Outpatient day cost at a primary hospital
Outpatient appointment cost per patient for watch and wait	$2.80	WHO-CHOICE	Outpatient day cost at a primary hospital
Inpatient day cost per patient at tertiary hospital	$12.90	WHO-CHOICE	Inpatient day cost at a tertiary hospital
Vital organ support cost per patient (entire duration of stay)	$1,658.90	Purba et al. 2020	Cost per surviving sepsis patient
Referral cost per patient	$12.81	Nanyonjo et al. 2015	
Length of stay for non-severe referred patients	3 days	Primary data	2-4 days

All patients were assumed to have an outpatient appointment cost for their initial assessment. Discharged patients had no additional costs. Referred patients with a non-severe outcome incurred a referral cost and an inpatient cost (daily cost multiplied by length of stay). Referred patients with a severe outcome incurred a referral cost and a vital organ support cost per patient (entire stay). Monitored patients with a non-severe outcome incurred an additional outpatient appointment cost. Monitored patients with a severe outcome incurred an additional outpatient appointment cost, referral cost, and a vital organ support cost per patient (entire stay).

15. Table S15: TRIPOD checklist.

Section/Topic			Checklist Item	Page
**Title and abstract**				
Title	1	D;V	Identify the study as developing and/or validating a multivariable prediction model, the target population, and the outcome to be predicted.	1
Abstract	2	D;V	Provide a summary of objectives, study design, setting, participants, sample size, predictors, outcome, statistical analysis, results, and conclusions.	3
**Introduction**				
Background and objectives	3a	D;V	Explain the medical context (including whether diagnostic or prognostic) and rationale for developing or validating the multivariable prediction model, including references to existing models.	4-5
	3b	D;V	Specify the objectives, including whether the study describes the development or validation of the model or both.	5
**Methods**				
Source of data	4a	D;V	Describe the study design or source of data (e.g., randomized trial, cohort, or registry data), separately for the development and validation data sets, if applicable.	17
	4b	D;V	Specify the key study dates, including start of accrual; end of accrual; and, if applicable, end of follow-up.	6
Participants	5a	D;V	Specify key elements of the study setting (e.g., primary care, secondary care, general population) including number and location of centers.	17
	5b	D;V	Describe eligibility criteria for participants.	17
	5c	D;V	Give details of treatments received, if relevant.	18
Outcome	6a	D;V	Clearly define the outcome that is predicted by the prediction model, including how and when assessed.	20
	6b	D;V	Report any actions to blind assessment of the outcome to be predicted.	NA
Predictors	7a	D;V	Clearly define all predictors used in developing or validating the multivariable prediction model, including how and when they were measured.	18; Table S2
	7b	D;V	Report any actions to blind assessment of predictors for the outcome and other predictors.	NA
Sample size	8	D;V	Explain how the study size was arrived at.	20; appendix p20
Missing data	9	D;V	Describe how missing data were handled (e.g., complete-case analysis, single imputation, multiple imputation) with details of any imputation method.	21
Statistical analysis methods	10a	D	Describe how predictors were handled in the analyses.	21
	10b	D	Specify type of model, all model-building procedures (including any predictor selection), and method for internal validation.	21
	10c	V	For validation, describe how the predictions were calculated.	22
	10d	D;V	Specify all measures used to assess model performance and, if relevant, to compare multiple models.	22
	10e	V	Describe any model updating (e.g., recalibration) arising from the validation, if done.	NA
Risk groups	11	D;V	Provide details on how risk groups were created, if done.	22
Development vs. validation	12	V	For validation, identify any differences from the development data in setting, eligibility criteria, outcome, and predictors.	7
**Results**				
Participants	13a	D;V	Describe the flow of participants through the study, including the number of participants with and without the outcome and, if applicable, a summary of the follow-up time. A diagram may be helpful.	6; Fig S1
	13b	D;V	Describe the characteristics of the participants (basic demographics, clinical features, available predictors), including the number of participants with missing data for predictors and outcome.	6-7; Table 1
	13c	V	For validation, show a comparison with the development data of the distribution of important variables (demographics, predictors and outcome).	6-7; Table 1
Model development	14a	D	Specify the number of participants and outcome events in each analysis.	6-7
	14b	D	If done, report the unadjusted association between each candidate predictor and outcome.	6-7
Model specification	15a	D	Present the full prediction model to allow predictions for individuals (i.e., all regression coefficients, and model intercept or baseline survival at a given time point).	Table S6
	15b	D	Explain how to the use the prediction model.	Table S6
Model performance	16	D;V	Report performance measures (with CIs) for the prediction model.	7-9; Table 2
Model-updating	17	V	If done, report the results from any model updating (i.e., model specification, model performance).	NA
**Discussion**				
Limitations	18	D;V	Discuss any limitations of the study (such as nonrepresentative sample, few events per predictor, missing data).	13-15
Interpretation	19a	V	For validation, discuss the results with reference to performance in the development data, and any other validation data.	14
	19b	D;V	Give an overall interpretation of the results, considering objectives, limitations, results from similar studies, and other relevant evidence.	12-13; 15-16
Implications	20	D;V	Discuss the potential clinical use of the model and implications for future research.	15-16
**Other information**				
Supplementary information	21	D;V	Provide information about the availability of supplementary resources, such as study protocol, Web calculator, and data sets.	25
Funding	22	D;V	Give the source of funding and the role of the funders for the present study.	23, 25
