# Supplementary material for: Predicting referral need for febrile children in low-resource community settings in South and Southeast Asia
Source: Nat Med. 2026 Apr 29;32(5):1907–16. doi: 10.1038/s41591-026-04338-1 (PMC13190324; doi:10.1038/s41591-026-04338-1)
Supplement: Supplementary file 2 — Reporting Summary [file 41591_2026_4338_MOESM2_ESM.pdf]

nature portfolio

Corresponding author(s): Arjun Chandna

Last updated by author(s): Feb 19, 2026

Reporting Summary

Nature Portfolio wishes to improve the reproducibility of the work that we publish. This form provides structure for consistency and transparency in reporting. For further information on Nature Portfolio policies, see our Editorial Policies and the Editorial Policy Checklist.

Statistics

For all statistical analyses, confirm that the following items are present in the figure legend, table legend, main text, or Methods section.

n/a	Confirmed	
☒	☐	The exact sample size (*n*) for each experimental group/condition, given as a discrete number and unit of measurement
☒	☐	A statement on whether measurements were taken from distinct samples or whether the same sample was measured repeatedly
☒	☐	The statistical test(s) used AND whether they are one- or two-sided<br>*Only common tests should be described solely by name; describe more complex techniques in the Methods section.*
☒	☐	A description of all covariates tested
☒	☐	A description of any assumptions or corrections, such as tests of normality and adjustment for multiple comparisons
☒	☐	A full description of the statistical parameters including central tendency (e.g. means) or other basic estimates (e.g. regression coefficient) AND variation (e.g. standard deviation) or associated estimates of uncertainty (e.g. confidence intervals)
☒	☐	For null hypothesis testing, the test statistic (e.g. *F*, *t*, *r*) with confidence intervals, effect sizes, degrees of freedom and *P* value noted<br>*Give P values as exact values whenever suitable.*
☒	☐	For Bayesian analysis, information on the choice of priors and Markov chain Monte Carlo settings
☒	☐	For hierarchical and complex designs, identification of the appropriate level for tests and full reporting of outcomes
☒	☐	Estimates of effect sizes (e.g. Cohen's *d*, Pearson's *r*), indicating how they were calculated

Our web collection on statistics for biologists contains articles on many of the points above.

Software and code

Policy information about availability of computer code

Data collection	Open Data Kit Collect version 1.25.1.
Data analysis	R version 4.3.2 and STATA version 18.

For manuscripts utilizing custom algorithms or software that are central to the research but not yet described in published literature, software must be made available to editors and reviewers. We strongly encourage code deposition in a community repository (e.g. GitHub). See the Nature Portfolio guidelines for submitting code & software for further information.

Data

Policy information about availability of data

All manuscripts must include a data availability statement. This statement should provide the following information, where applicable:

- Accession codes, unique identifiers, or web links for publicly available datasets
- A description of any restrictions on data availability
- For clinical datasets or third party data, please ensure that the statement adheres to our policy

De-identified, individual participant data from this study will be available to researchers whose proposed purpose of use is aligned with the study objectives and approved by the data access committees at MSF and MORU. Enquiries or requests for data can be sent to data.sharing@london.msf.org and datasharing@tropmedres.ac, with an anticipated timeline of one month between submission of a request and committee decision. Researchers interested in

accessing biobanked samples should contact the corresponding author, who will coordinate with the Spot Sepsis Sample Use Committee, with an anticipated timeline of one month between submission of a request and committee decision.

Research involving human participants, their data, or biological material

Policy information about studies with human participants or human data. See also policy information about sex, gender (identity/presentation), and sexual orientation and race, ethnicity and racism.

Reporting on sex and gender	Proportion of male and female participants recruited reported in Table 1
Reporting on race, ethnicity, or other socially relevant groupings	No reporting on race or ethnic groups performed as these vary considerably across and within different study locations
Population characteristics	3,423 children aged 1-59 months presenting with acute febrile illnesses. Screening occurred during daytime working hours. Inpatients were recruited consecutively. A random selection of outpatients were recruited, informed by computer-generated random number tables which utilised the previous week's routinely collected hospital data for the sampling frame. The main biases that may have occurred as a result of the recruitment process is that the study sample may not be representative of patients presenting out of hours and that the outpatient sample may be more representative of patients attending the hospital earlier in the working day.
Recruitment	5 March 2020 to 4 November 2022
Ethics oversight	The study received ethical approval from the sponsors (Médecins Sans Frontières Ethical Review Board MSF ERB 1967; Oxford Tropical Medicine Research Committee OxTREC 59-19) and ethical review boards in all participating countries (International Centre for Diarrhoeal Disease Research, Bangladesh PR-200006; Angkor Hospital for Children Research Committee, Cambodia 01296/AHC; National Ethics Committee for Health Research, Cambodia 264/NECHR; Medical and Health Research Ethics Committee, Indonesia KE/FK/1397/EC/2019; National Ethics Committee for Health Research, Laos 051/NECHR; University of Medicine and Pharmacy at Ho Chi Minh City, Viet Nam; 818/HDDD-DHYD; Ethics Committee for Biomedical Research, Viet Nam VNCH-RICH-2021-77).

Note that full information on the approval of the study protocol must also be provided in the manuscript.

Field-specific reporting

Please select the one below that is the best fit for your research. If you are not sure, read the appropriate sections before making your selection.

☒ Life sciences ☐ Behavioural & social sciences ☐ Ecological, evolutionary & environmental sciences

For a reference copy of the document with all sections, see nature.com/documents/nr-reporting-summary-flat.pdf

Life sciences study design

All studies must disclose on these points even when the disclosure is negative.

Sample size	The methods of Riley et al. were followed and using an anticipated outcome prevalence of 1%, conservative R2 Nagelkerke of 0.15, and shrinkage factor of 0.9, it was estimated that six events per parameter would be required to derive the prediction models. The Covid-19 pandemic, declared in the same week that recruitment began at the first site, delayed initiation of other sites and slowed enrolment. Study duration (and thus sample size) was determined by available resources, with recruitment continuing for as long as funding allowed. Accordingly, as there were 97 events in the derivation dataset, a maximum of 16 candidate parameters could be included for development of the prediction models, whilst minimising the risk of overfitting.
Data exclusions	18 participants. Lost to follow up and so no data available available on outcome status.
Replication	Cross-validation during the development of the prediction models. External validation of the prediction models in a held-out dataset.
Randomization	Not applicable - observational study.
Blinding	Not applicable - observational study therefore no group allocation.

Reporting for specific materials, systems and methods

We require information from authors about some types of materials, experimental systems and methods used in many studies. Here, indicate whether each material, system or method listed is relevant to your study. If you are not sure if a list item applies to your research, read the appropriate section before selecting a response.

Materials & experimental systems

n/a	Involved in the study
☒	☐ Antibodies
☒	☐ Eukaryotic cell lines
☒	☐ Palaeontology and archaeology
☒	☐ Animals and other organisms
☐	☒ Clinical data
☒	☐ Dual use research of concern
☒	☐ Plants

Methods

n/a	Involved in the study
☒	☐ ChIP-seq
☒	☐ Flow cytometry
☒	☐ MRI-based neuroimaging

Clinical data

Policy information about clinical studies

All manuscripts should comply with the ICMJE guidelines for publication of clinical research and a completed CONSORT checklist must be included with all submissions.

Clinical trial registration: ClinicalTrials.gov NCT04285021

Study protocol: https://osf.io/v594s/ and https://bmjopen.bmj.com/content/11/1/e045826

Data collection:
Goyalmara Mother and Child Hospital, Bangladesh: 18/03/2021 to 27/04/2022
Angkor Hospital for Children, Cambodia: 05/03/2020 to 24/02/2022
Rumah Sakit Umum Daerah Wates, Indonesia: 22/03/2021 to 22/04/2021
Salavan Provincial Hospital, Lao PDR: 10/09/2020 to 30/08/2021
Savannakhet Provincial Hospital, Lao PDR: 21/01/2021 to 26/08/2021
Dong Nai Children's Hospital, Viet Nam: 10/05/2021 to 28/10/2022
Viet Nam National Children's Hospital, Viet Nam: 08/12/2021 to 04/11/2022

Outcomes:
Primary outcome: death or receipt of organ support (mechanical ventilation, non-invasive ventilation, inotropic therapy, or renal replacement therapy) within two days of enrolment. Assessed by research staff via observation at the bedside and/or interview with caregiver.

Secondary outcome: Category IV = Death or organ support ≤ 2 days after enrolment; Category III = Admission to any health facility for > 2 nights between enrolment and D28 OR death or organ support between D2 and D28; Category II = Admission to any health facility for ≤ 2 nights between enrolment and D28 OR symptoms not resolved by D28; Category I = Not admitted to any health facility between enrolment and D28 AND recovered by D28. Assessed by research staff via observation at the bedside and/or interview with caregiver.

Plants

Seed stocks: NA

Novel plant genotypes: NA

Authentication: NA
